# Risk and Outcome after Ablation of Isthmus-Dependent Atrial Flutter in Elderly Patients

**DOI:** 10.1371/journal.pone.0127672

**Published:** 2015-05-22

**Authors:** Béatrice Brembilla-Perrot, Jean Marc Sellal, Arnaud Olivier, Vladimir Manenti, Thibault Villemin, Daniel Beurrier, Christian De Chillou, Zohra Lamiral, Nicolas Girerd

**Affiliations:** 1 Department of Cardiology, Nancy University Hospital, Vandoeuvre-les-Nancy, France; 2 INSERM, Centre d’Investigations Cliniques 1433, Université de Lorraine, Institut Lorrain du cœur et des vaisseaux, CHU de Nancy, Nancy, France; Scuola Superiore Sant'Anna, ITALY

## Abstract

**Purpose of the research:**

To study the influence of age on the clinical presentation and long-term outcome of patients referred for atrial flutter (AFL) ablation. Age-related differences have been reported regarding the prognosis of arrhythmias.

**Methods:**

A total of 1187 patients with a mean age 65±12 years consecutively referred for AFL ablation were retrospectively analyzed in the study.

**Results:**

445 (37.5%) patients were aged ≥70 (range 70 to 93) among which 345 were aged 70 to 79 years (29.1%) and 100 were aged ≥80 (8.4%). In multivariable analysis, AFL-related rhythmic cardiomyopathy and presentation with 1/1 AFL were less frequent (respectively adjusted OR = 0.44, 0.27–0.74, p = 0.002 and adjusted OR = 0.29, 0.16–0.52, p<0.0001). AFL ablation-related major complications were more frequent in patients ≥70 although remained lower than 10% (7.4% in ≥70 vs. 4.2% in <70, adjusted OR = 1.74, 1.04–2.89, p = 0.03). After 2.1±2.7 years, AFL recurrence was less frequent in patients ≥70 (adjusted OR = 0.54, 0.37–0.80, p = 0.002) whereas atrial fibrillation (AF) occurrence was as frequent in the 70–79 and ≥80 age subsets. As expected, cardiac mortality was higher in older patients. Patients aged ≥80 also had a low probability of AFL recurrence (5.0%) and AF onset (19.0%).

**Conclusions:**

Older patients represent 37.5% of patients referred for AFL ablation and displayed a <10% risk of ablation-related complications. Importantly, AFL recurrences were less frequent in patients ≥70 while AF occurrence was as frequent as in patients <70. Similar observations were made in patients ≥80 years. AFL ablation appears to be safe and efficient and should not be ruled out in elderly patients.

## Introduction

Atrial flutter (AFL) is a frequent condition [1]. Because of its feasibility, effectiveness and low procedural risk, radiofrequency (RF) ablation can be performed as a first line treatment of AFL [2–6]. As a result, most patients presenting with AFL in clinical contexts other than acute treatable conditions are now often treated with RF cavotricuspid valve isthmus ablation.

Age-related differences have been reported for arrhythmias [7, 8]. Moreover, elderly patients more frequently present with atrial fibrillation (AF), thromboembolic events and ablation-related complications [9] and, therefore, often do not benefit from the curative RF ablation of AFL.

The purpose of this retrospective study was to investigate the influence of age on the clinical presentation and long-term outcome of patients referred for AFL ablation.

## Methods

### Population

From January 1999 to March 2014, 1276 patients were referred to our institution for RF ablation of a documented recurrent AFL or a poorly-tolerated first episode of AFL.

The main diagnostic criterion for AFL was visible and highly regular “F” waves at a rate ≤350 bpm. Highly regular “F” waves were defined as those in which the cycle to cycle atrial variability was ≤10 ms. “F” wave rates had to be greater than 190 bpm among patients receiving class I or class III antiarrhythmic agents and greater than 240 bpm otherwise. AFL was counterclockwise in 937 patients (79%) and clockwise in 250 patients (21%).

Eighty-nine patients were excluded from the present study because AFL was proven to be isthmus-independent during the electrophysiological procedure, among whom 3 patients had previously undergo left atrial ablation. Hence, a total of 1187 consecutive patients, 908 males and 279 females, with isthmus-dependent AFL were included in the study.

### Protocol

#### Data collection

Prior medical history, prior thromboembolic events, diabetes, symptoms, previous medications including the prescription of antiarrhythmic drugs (AAD), history of AF and physical examination findings were obtained from the clinical records of each patient.

Hypertension was defined as self-reported history of hypertension associated with the use of antihypertensive medication or, in untreated patients, as a blood pressure at rest greater than 140/90 mmHg. Coronary artery disease was defined as a documented history of myocardial infarction and/or coronary revascularization, or the presence of one or more significant (>70%) obstructive lesion(s) on a prior coronary angiogram. Patients were considered to have valvular heart disease if they suffered from moderate to severe valvular regurgitation or mitral stenosis, regardless of severity.

This monocenter retrospective study on patients’ files was approved by the Commission Nationale Informatique et Libertés (CNIL). Informed written consent was not required by regulation. Patients’ data was anonymized and de-identified prior to analysis.

#### Electrophysiological procedures protocol

Ablation of AFL by RF was performed after signed informed consent by a conventional method using a multipolar catheter placed at the coronary sinus whose poles recorded the respective activity at the coronary sinus isthmus and lateral wall of the right atrium. Energy was delivered by an 8 mm tip RF catheter with a maximum power of 70 W and a maximum target temperature of 70 degrees Celsius. Four senior operators in association (or not) with fellow cardiologists performed the large majority of the ablations. Details of the protocol have previously been described [10–12]. Ablation was performed after analgesia by infusion of 5 mg of Nalbuphine.

Among patients with permanent AFL, sinus rhythm was obtained by applying the RF current at the cavotricuspid right isthmus. Successful ablation in this isthmus was the main factor confirming the presence of a typical isthmus-dependent flutter. Recording of discrete split potentials along the length of the crista terminalis suggested the presence of conduction block. In patients in whom atrial flutter was not interrupted by RF current after 20 applications, entrainment mapping was used and concealed entrainment from the low right atrial isthmus was demonstrated in order to establish isthmus-dependent flutter. Obtaining an isthmus block was the next objective and was achieved and verified in patients in sinus rhythm. The ablation was considered to be successful when a bidirectional isthmus block could still be verified at least 20 minutes after the last RF application.

Ablation-related complications were recorded. Major complications were defined as life-threatening arrhythmia, with either poor hemodynamic tolerance or syncope, and requiring urgent treatment. These exceptional complications have recently been reported by our group [10].

#### Pharmacological management after the electrophysiological procedures

As a general rule, antiarrhythmic drugs were discontinued in patients without AF history and maintained in patients with a history of AF. Of note, in patients without AF history, beta-blockers were maintained when patients had indications other than AFL for beta-blocker medication (i.e. ischemic heart disease, dilated cardiomyopathy or hypertension). Anticoagulants were maintained in patients with a history of AF based on the following criteria: 1) concomitant heart disease before 2010, 2) CHADS2 scores ≥1 between 2010 and 2012, and 3) CHA2DS2-VASc ≥1 after 2012.

#### Follow-up

Patients were followed up for a mean of 2.1±2.7 years by the referring cardiologist. ECG and 24-hour Holter monitoring were systematically performed at one month after AFL ablation and once yearly thereafter. ECG and 24-hour Holter recordings were also performed if the patient reported palpitations or symptoms suspected to be the consequence of AF or AFL occurrence/recurrence.

### Statistical methods

Continuous variables are expressed as mean±SD and were compared using t tests for independent samples or ANOVA as appropriate. Differences in proportion were compared using a Chi^2^ test.

Logistic regression with age <70 years and age ≥70 years were used as dependent variables. All variables associated with a p-value <0.20 on univariable logistic regression analysis were considered as candidate adjustment covariates in the multivariable models. A backward selection process which only retained gender and previous heart disease as adjustment covariates was then used.

A p value <0.05 was considered statistically significant. All statistical analyses were performed using the SPSS package for Windows (version 21, IBM Corp., Armonk, NY, USA).

## Results

### Age distribution of the population

Of the 1187 patients studied, 445 (37.5%) patients were ≥70 (range 70 to 93) among which 345 were aged 70 to 79 years (29.1%) and 100 aged ≥80 (8.4%). The remaining 742 patients were under 70 years of age (62.5%).

### Clinical data according to age subsets

Heart disease (HD) was more frequent in older patients (82.2% vs. 74.0%, p = 0.001, Table 1). Previous atrial fibrillation (AF) was as frequent in patients <70 and ≥70 (32.5% vs. 31.9%, p = 0.84). Older patients tended to receive less antiarrhythmic drugs and/or beta-blockers than patients <70 (63.3% vs. 68.8%, p 0.06). AFL-related tachycardiomyopathy and 1/1 AFL were less frequent in patients ≥70 than in patients <70 (4.7% vs. 9.3%, p<0.004 and 3.1% vs. 10.2%, p<0.001 respectively).

**Table 1:** Clinical characteristics according to age subsets.

	Patients aged<70 (N = 742)	Patients aged ≥70 (N = 445)				
		70–93 (N = 445)	p value <70 vs >70	70–79 (N = 345)	80–93 (N = 100)	p value <70 vs 70–79 vs 80–93
**Age (years)**	58±9	76.5±5		74±3	83±3	
**Male gender**	578 (77.9%)	330 (74.2%)	0.14	266 (77.1%)	64 (64.0%)	0.01
**Previous heart disease**	549 (74.0%)	366 (82.2%)	0.001	284 (82.3%)	82 (82.0%)	0.05
**History of atrial fibrillation**	241 (32.5%)	142 (31.9%)	0.84	104 (30.1%)	38 (38.0%)	0.32
**History of stroke**	21 (2.8%)	18 (4.0%)	0.26	14 (4.1%)	4 (4.0%)	0.52
**Diabetes**	87 (11.7%)	61 (13.7%)	0.32	48 (13.9%)	13 (13.0%)	0.59
**Antiarrhythmic drugs, betablockers**	470 (63.3%)	306 (68.8%)	0.06	235 (68.1%)	71 (71.0%)	0.14
**Rhythmic cardiomyopathy**	69 (9.3%)	21 (4.7%)	0.004	16 (4.6%)	5 (5.0%)	0.02
**1/1 atrial flutter**	76 (10.2%)	14 (3.1%)	< 0.0001	11 (3.2%)	3 (3%)	<0.001

### Procedural and post-procedural events according to age subsets

A higher proportion of patients ≥70 had ablation failure, although the proportion difference across groups did not reach statistical significance (Table 2, p = 0.11). In contrast, AFL ablation-related complications were more frequent in patients ≥70 than in patients <70 (p = 0.02). The proportion of minor and major complications was similar in patients 70 to 79 and in patients ≥ 80 (7.5% and 7.0%, respectively). These complications were either major, defined as those that resulted in permanent injury or death, required an interventional remedy, or prolonged the duration of hospitalization (n = 16) or minor (n = 48). Major complications consisted in poorly tolerated bradycardia (transient complete atrioventricular block or sinus bradycardia <40 bpm, n = 10, associated in 5 cases with cardiac shock and acute renal failure in 5 patients), tamponade (n = 1), bleeding leading to death (n = 1), various AE-related deaths (n = 2), ventricular tachycardia-related death (n = 1), and right coronary artery occlusion-related complete atrioventricular block (n = 1). These major complications occurred in 8 patients ≥70 (2%) and 8 patients <70 (1.1%) (p = 0.3). Moderate and minor complications were transitory major sinus bradycardia or second- or third-degree atrioventricular block (n = 40), bleeding (n = 4), transient ischemic attack (n = 1), and various AE (n = 3). Minor complications occurred in 25 patients ≥70 (5.6%) and 23 patients <70 (3%) (p = 0.03). AVB-related ablation requiring a pacemaker implantation was rare (1 patient <70 and 1 ≥70).

**Table 2:** Procedural and post-procedural events according to age subsets.

	Patients aged<70 (N = 742)	Patients aged ≥70 (N = 445)				
		70–93 (N = 445)	p value <70 vs >70	70–79 (N = 345)	80–93 (N = 100)	p value<70 vs 70–79 vs 80–93
**Ablation failure**	71 (9.6%)	56 (12.6%)	**0.11**	42 (12.2%)	14 (14.0%)	0.24
**Complications**	31 (4.2%)	33 (7.4%)	**0.02**	26 (7.5%)*	7 (7%)	0.06
**Atrial flutter recurrence**	110 (14.8%)	40 (9.0%)	0.003	35 (10.1%)*	5 (5.0%)*	0.005
**Atrial fibrillation after ablation**	173 (23.3%)	100 (22.5%)	0.74	81 (23.5%)	19 (19.0%)	0.61
**Death**	37 (5.0%)	41 (9.3%)	0.004	34 (9.9%)	7 (7.0%)	0.01
**Pacemaker**	45 (6.1%)	67 (15.1%)	<0.001	50 (14.5%)	17 (17.0%)	<0.001
**His ablation**	5 (0.7%)	12 (2.7%)	0.005	8 (2.3%)	4 (4.0%)	0.008
**Atrial fibrillation ablation**	38 (5.1%)	0 (0.0%)	<0.001	0 (0.0%)	0 (0.0%)	<0.001

AFL recurrences were less frequent in patients ≥70 than in patients <70 (9.0% vs. 14.8%, p = 0.003) whereas the risk for AF did not significantly differ between the two age subsets (22.5% vs. 23.3%, p = 0.74).

As expected, mortality was significantly higher in older patients (p = 0.004). In addition, the management of arrhythmias recurrences also differed significantly according to age. Pacemaker implantation and His bundle ablation (n = 17) were performed in patients with rapid AF despite the use of bradycardic agents. This strategy was chosen in patients >75 or in younger patients with comorbidities which contra-indicated AF ablation. AF ablation was never performed in older patients.

### Univariable and multivariable analysis of clinical presentations and clinical outcome (Table 3)

Associations between age ≥70 years vs. <70 years in univariable and multivariable logistic regression adjusted for previous HD and gender yielded similar results. Rhythmic cardiomyopathy and 1/1 AFL were markedly less frequent in patients ≥70 (OR = 0.44, 0.27–0.74, p = 0.002 and OR = 0.29, 0.16–0.52, p<0.001 respectively in the multivariable model, Table 3). Patients ≥70 had a 1.7-fold higher risk of AFL ablation-related complications (Table 3) but had a markedly lower risk for AFL recurrence (OR = 0.54, 0.37–0.80, p = 0.002).

**Table 3:** Associations between age (>70 vs<70) in univariable and multivariable logistic regression with clinical presentation and outcomes.

	Univariable logistic model			Multivariable logistic model*		
	OR for age>70 vs<70	CI	P	OR for age>70 vs<70	CI	p
Rythmic cardiomyopathy	0.48	0.29–0.80	0.005	0.44	0.27–0.74	0.002
1/1 atrial flutter	0.29	0.16–0.51	<0.001	0.29	0.16–0.52	<0.001
Complication	1.84	1.11–3.04	0.02	1.74	1.04–2.89	0.03
Atrial flutter recurrence	0.57	0.39–0.83	0.004	0.54	0.37–0.80	0.002
Atrial fibrillation after Atrial flutter ablation	0.95	0.72–1.26	0.74	0.92	0.70–1.23	0.58
Pacemaker implantation	2.75	1.84–4.09	<0.001	2.56	1.71–3.82	<0.001

*Model adjusted on previous heart disease and gender.

## Discussion

The main findings of this study are that patients aged 70 or older 1) were less likely to have a severe form of AFL such as rhythmic cardiomyopathy or AFL with 1:1 ventricular conduction at the time of AFL ablation, 2) had a similar success probability of the ablation but a slightly less than 2-fold higher probability of procedural complication and 3) had a very good post-procedural success of AFL ablation, including a low AFL recurrence rate. Similar results were also observed when restricting the analysis to patients 80 or older.

AF is a common arrhythmia in elderly persons and a common cause of embolic stroke [7]. Advanced age is the main predictor of AF prevalence [7] and occurrence in a variety of clinical situations [13]. A similar association with age is observed with AFL, and AFL and AF are often associated [12, 14–23]. AFL ablation in patients with either isolated AFL or AFL associated with paroxysmal AF remains the preferred method of treatment [24]. Yet, while age is the main determinant of the risk for AFL, AFL ablation is often primarily performed in younger patients.

In our cohort, the risk of AFL-related complications was higher in older patients. Indeed, we previously reported a higher incidence of such complications during AFL ablation in older patients, many of which were related to the drugs used to slow the rate of atrial flutter [10]. The abrupt changes in rates from a rapid rate in atrial flutter to a sinus bradycardia could moreover be associated with cardiogenic shock or acute renal failure. These data differ from those of Bohnen and al. [9] who reported that aside from ablation type, renal insufficiency was the only independent predictor of a major complication, whereas age, gender, body mass index, international normalized ratio level, hypertension, coronary artery disease, diabetes, and prior cerebrovascular accident were not associated with increased risk. In the present study, renal function was not known for all patients; we report a less than 4% absolute increase in the risk for complications in patients 70 or older, which is seemly quite moderate.

More importantly, while we did observe a moderately higher complication probability in elderly patients, we also identified a greater benefit of ablation in these patients. Indeed, older patients experienced a similar AF probability and lower AFL recurrence probability after AFL ablation, with a 5% absolute risk reduction for AFL recurrence in patients 70 or older, and a 10% absolute risk reduction in patients 80 or older. Given this higher treatment effect observed in elderly patients, the benefit to risk ratio of AFL ablation would thus appear positive despite a higher complication probability in this population.

Pacemaker implantation and His bundle ablation were also found to more frequently used in the elderly patients. In addition, AF ablation was not performed in this population of patients. However, given that several studies have recently suggested that outcomes of catheter ablation for AF in the elderly can be just as successful as in younger patients [25], this procedure could henceforth be performed more frequently in this population in the near future, as witnessed by the increasing number of reports addressing AF and AFL ablations [26–28].

### Clinical perspectives

Age is the main predictor of the incidence of AFL and AF as well as a key contributor to the risk of stroke in these patients [29]. In the CHA2DS-VAsc scoring system, age >75 is assigned a rating of 2 points, of equal importance to that of stroke. However, it is very probable that elderly patients are undertreated for their AFL, as observed for several other pathologies in the cardiovascular field [30, 31]. It has already been reported than elderly patients are undertreated with oral anticoagulants despite their high thromboembolic risk [32]. As observed with AF radiofrequency [33], it is very likely that elderly patients are also undertreated with AFL radiofrequency, in spite of the fact that several studies have demonstrated that arrhythmia ablation, including AF radiofrequency, is safe even in elderly patients [33–35].

The safety of AFL radiofrequency, which is much simpler than AF radiofrequency, has been insufficiently studied. The present findings clearly demonstrate than elderly patients have a low complication risk, which should promote the referral of elderly patients with AFL to AFL radiofrequency. This referral should also be promoted in light of the excellent efficacy results of the procedure. Of key importance, the low risk of AFL recurrence and AF occurrence identified herein in older patients after AFL ablation could translate in noteworthy lower stroke rates in this population at high risk for stroke.

### Limitations

Given that patients with AFL in this study were referred for catheter ablation, our results cannot be extended to the entire population of patients with AFL. Mean follow-up was limited to 2.1 years and hence, longer-term occurrence of arrhythmia risk cannot be ruled out, particularly in younger patients as they have a lower risk of competing events. The low event rate for AF may reflect the short follow-up and methodology used to detect atrial arrhythmias after ablation for flutter. Systematic Holter monitoring was performed only once a year during follow-up. This annual Holter monitoring results in a fairly low probability of detecting asymptomatic episodes of paroxysmal AF.

## Conclusion

In our hands, older patients exhibited a <10% risk of ablation-related complications, even in the subset of patients ≥80 years. Importantly, AFL recurrences were less frequent in patients ≥70 while AF occurrence was as frequent as that documented in patients under 70. Similar observations were made in patients ≥80 years. Hence, as elderly patients are the most at risk of stroke related to AFL and AF, AFL ablation appears to be a safe and efficient interventional procedure in elderly patients and should thus be encouraged in this particular age group.
